# Diagnostic, therapeutic and evolutionary characteristics of cervical cancer in Department of Radiotherapy, Mohamed V Military Hospital – Rabat in Morocco

**DOI:** 10.1186/s40661-015-0009-y

**Published:** 2015-07-27

**Authors:** Mohammed Elmarjany, Abdelhak Maghous, Rachid Razine, Elamin Marnouche, Khalid Andaloussi, Amine Bazine, Issam Lalya, Noha Zaghba, Khalid Hadadi, Hassan Sifat, Baba Habib, Jaouad Kouach, Hamid Mansouri

**Affiliations:** Department of Radiotherapy, Mohamed V Military Hospital, Rabat, Morocco; National Institute of Oncology, Rabat, Morocco; Department of Public Health, Laboratory of Biostatics, Clinical Research and Epidemiology, School of Medicine and Pharmacy of Rabat, Rabat, Morocco; Department of Gynecology, Mohamed V Military Hospital, Rabat, Morocco

**Keywords:** Cancer of uterine cervix, Radiotherapy, Concomitant radio-chemotherapy

## Abstract

**Background:**

Cancer of uterine cervix is the second most common cause of cancer related deaths among women. The aim of this study is to report the experience of Military Hospital Mohamed V in the management of cervical cancer and their results.

**Methods:**

All cervical cancer managed at the radiotherapy department of Military Hospital Mohamed V between January 2005 and February 2010, were included for investigation of their demographic, histological, therapeutic and follow-up characteristics. Of the 162 cases managed, 151 (93.2 %) cases were treated in our department.

**Results:**

In our study the median age was 51.5 years (33–82). The median duration of symptoms before diagnosis was four [3, 7] months. The major presenting complaints were abnormal vaginal bleeding (89.8 %). Squamous cell carcinoma cervix was seen in 86.2 % (*n* = 137), adenocarcinoma in 11.3 % (*n* = 18) and adenosquamous carcinoma in 2.4 % (*n* = 4). One hundred seventeen (84.8 %) cases were seen at late stage. An abdominal and pelvic computed tomography (CT) scan was performed in 34.6 % (*n* = 56) of cases, magnetic resonance imaging (MRI) in 62.9 % (*n* = 102). The pelvic lymph nodes were achieved in 16.6 % of cases.

Over half of patients 58.3 % (*n* = 88) were treated with a combination of external beam radiation therapy (EBRT) and a concurrent cisplatin based chemotherapy (40 mg /m2 weekly).

With a mean of 51.6 months (2 to 109), we recorded 19 (12.6 %) pelvic relapse and 15 (9.9 %) metastases. The median time to onset was 19.4 months (2–84 months). The local control rate was 63.6 % (*n* = 96) and 21 (13.9 %) patients were lost to follow-up. The overall survival (OS) at 3 years and 5 years was respectively 78.3 % and 73.6 % and the relapse-free survival (RFS) was respectively 80 % and 77.2 %.

**Conclusion:**

Most of cervical cancer patients in Morocco are seen at late stage necessitating referral for radiotherapy, chemotherapy or palliative care. This may reflect lack of cervical screening in order to early detect and treat pre-malignant disease stage.

## Background

Cervical cancer is the second most common cancer in women worldwide [1]. It is estimated that there are more than 529 000 new cases diagnosed annually and more than 274,000 deaths in the world in 2008, of which more than 87 % occur in developing countries [2, 3]. Cervical cancer continues to be related to socio-economic and demographic (SEDS) disparities in both developing and developed countries. In the U.S., although the overall downward trend in cervical cancer there still exists a disproportion in mortality rates for cervical cancer related deaths among ages, racial, geographic and socio-economic groups [4, 5]. Analyses of the USA cancer data have shown that mortality due to cervical cancer increases with poverty and decreasing education [6]. Studies have publicized that late stage at diagnosis is correlated with lower survival rates [7]. It has also been reported that longer durations of symptoms as well as of treatment prolongation negatively affect survival [8, 9]. These two factors can therefore be useful predictors for the severity of illness and likelihood of survival.

In Morocco, cervical cancer is the second most common cancer of women after breast cancer [10]. According to GLOBOCAN 2008 [2], the world age-standardized incidence of cervical cancer among women in Morocco was 14.1 new cases/100 000 inhabitants/year (1979 new cases/year). The mortality rate from this cancer was 8.4 per 100 000. The stage of diagnosis is the most important independent factor of prognosis [11, 12]. In Morocco, a recent study [13] showed a late diagnosis of cervical cancer. Indeed, 43.7 % were presented with stage II at diagnosis (FIGO) and 38.1 % were presented in advanced stages (stages III and IV).

The aim of this study is to report the experience of Military Hospital Mohamed V in the management of cervical cancer, the various treatment modalities used and their results.

## Methods

This is a retrospective analytic study of 162 cervical cancer patients managed at the radiotherapy department of Military Hospital Mohamed V of Rabat in Morocco, between January 2005 and February 2010.

Data was collected using a well structured checklist containing important study parameters. The record collection includes patient demographic data (age of diagnosis, of marriage and age of first pregnancy (years)), menopausal status (premenopausal or postmenopausal) and parity was separated on three groups (0, 1–4 and ≥ 5). Data includes also clinical presenting symptoms (abnormal vaginal bleeding, offensive vaginal discharge, pelvis pain…), duration of symptoms (months), vaginal invading (free, upper, medium or lower vaginal wall) and other clinical data such as classification FIGO of disease (IA, IB, IIA, IIB, IIIB, IVA, IVB), histological type (squamous, adenocarcinoma or adenosquamous), pelvic and/or lateral aortic adenopathy, parametrial invasion, date of primary diagnosis. The type and modalities of primary treatment (radiotherapy, chemotherapy, both or/and surgery), date and sites of relapse (pelvic and/or metastases), the follow up data, death date and date of last follow-up visit were also recorded.

Statistical analysis of the data was carried out by the SPSS for Windows (SPSS, Inc., Chicago, IL, USA). Qualitative variables were presented as number and percentages. Quantitative variables were presented as mean ± standard deviation for variables with normal distribution, and as median and interquartile range (IQR) for variables with skewed distributions. The survival rate was analyzed with the Kaplan-Meier method.

## Results

Of the 162 cases of cervical cancer managed, 151 (93.2 %) cases were treated in our department. Table 1 shows their epidemiological characteristics. Their ages ranged from 33 to 82 years old with a mean of 51.5 (±11.5) years. More than half of patients were post menopausal 85 (52.5 %) and married early 58 (63.7 %). Age of first pregnancy ranged from 14 to 36 with a mean of 20.3 (±4.2). Also, their parity ranged from 0 to 12 with a mean of 4.9 (±2.6).

**Table 1:** Clinical and para-clinical epidemiological characteristics (*n* = 162)

Item	Frequency (%)	Range	Mean (±standard deviation)
Age of diagnosis (years)		33-82	51.5 (±11.5)
≤39	21 (12.9)		
40–49	61 (37.6)		
50–59	45 (27.7)		
60–69	18 (11.1)		
≥70	17 (10.5)		
Menopausal status			
Premenopausal	77 (47.5)		
Postmenopausal	85 (52.5)		
Age of marriage (years)		12-30	17.9 (±3.6)
≤18	58 (63.7)		
>18	33 (36.3)		
Age of first pregnancy (years)		14-36	20.3 (±4.2)
Parity		0-12	4.9 (±2.6)
0	5 (3.7)		
1-4	65 (47.8)		
≥5	66 (48.5)		
Duration of symptoms (months)			4 [3, 7]
Presenting complaints			
Abnormal vaginal bleeding	143 (89.9)		
Offensive vaginal discharge	91 (57.2)		
Pelvic pain	44 (27.7)		
Haematuria	2 (1.3)		
Clinically review			4.8 (±1.6)
Tumor size (cm)			
Vaginal invading			
Upper	64 (57.7)		
Medium	15 (13.5)		
Lower	4 (3.6)		
Free	28 (25.2)		
Parametrial invasion	95 (67.4)		
Histological type			
Squamous cell carcinoma	137 (86.2)		
Adenocarcinoma	18 (11.3)		
Adenosquamous carcinoma	4 (2.5)		
Para clinical review			
Abdominal and pelvic CT	56 (34.6)		
Pelvic adenopathy	12 (21.4)		
Lateral aortic adenopathy	1 (1.8)		
Pelvic MRI			41.6 (±16.8)
Tumor size (mm)	102 (93)		
Pelvic adenopathy	16 (15.7)		
Parametrial invasion	57 (55.9)		
Hemoglobin (g/dl)			11.8 (±1.8)

Abnormal vaginal bleeding was the most common symptom reported by 143 (89.9 %) patients. Other symptoms were offensive vaginal discharge 91 (57.2) and pelvic pain 44 (27.7). The median duration of symptoms before diagnosis was four months [3, 7]. Tumor size was 4.8 cm (±1.6) clinically and in 64 (57.7 %) patient’s upper vaginal wall was invaded. Squamous cell carcinoma 137 (86.2 %) was the leading histological type, whereas adenocarcinoma contributed 18 (11.3 %) and 4 (2.5 %) were adenosquamous carcinoma.

An abdominal and pelvic CT was performed in 34.6 % (*n* = 56) of cases, MRI in 62.9 % (*n* = 102). The pelvic lymph nodes were achieved in 16.6 % of cases. In MRI Tumor size was 41.6 (±16.8) mm and parametrial was invaded in 57 (55.9 %) of cases.

**Fig. 1:**
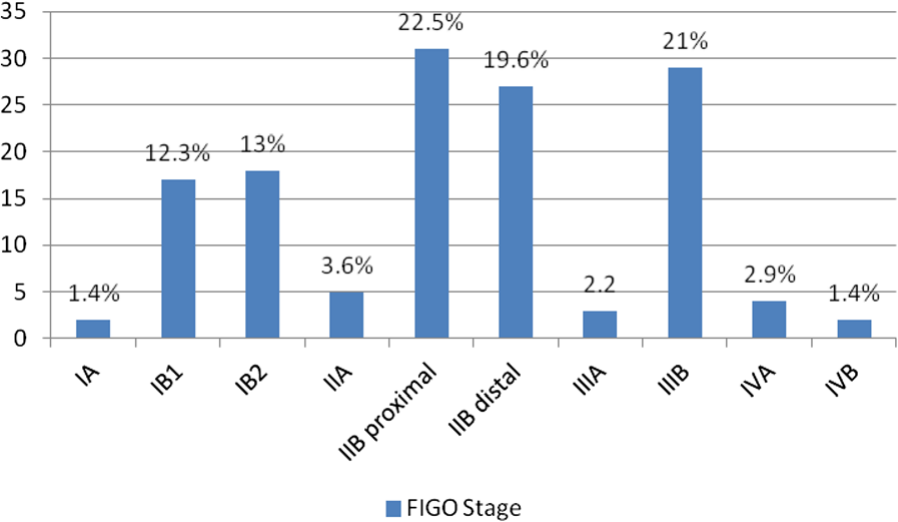
FIGO stage of disease

Based on International Federation of Gynaecology and Obstetrics classification for staging cervical cancer (ACS, 2008), 2 (1.4 %), 17 (12.3 %), 18 (13 %), 5 (3.6 %), 31 (22.5 %), 27 (19.6 %), 3 (2.2 %), 29 (21 %), 4 (2.9 %) and 2 (1.4 %) of cases, cancer stages were respectively IA, IB1, IB2, IIA, IIB proximal, distal IIB, IIIA, IIIB, VIA and VIB (Fig. 1).

One hundred fifty one (93.2 %) cases were treated in our department. Table 2 shows their modalities. Over half of patients 58.3 % (*n* = 88) were treated with a combination of external beam radiation therapy (EBRT) and a concurrent cisplatin based chemotherapy (40 mg /m2 weekly). Fifty (34 %) patients underwent surgery as their initial treatment. Forty six (31.4 %) of these received post operative radiotherapy or concomitant radio-chemotherapy following surgery due to positive pelvic lymphnodes, narrow or positive surgical margins or other poor risk factors. The surgical procedure was Piver II; a modified radical hysterectomy with annexectomy, which includes removal of the uterus, cervix, upper one-fourth of the vagina, and parametria. The surgeon also performs a bilateral pelvic lymphadenectomy via laparotomy in 57 (85.1 %) cases, who brought 4.7 ± 3.9 nodes on the right and 4.1 ± 3.7 nodes on the left, of which was only invaded in 18/471 (3.82 %). Conventional laparoscopy or robot-assisted laparoscopy was not available in our hospital.

**Table 2:** Therapeutic modalities (*n* = 151)

Therapeutic modalities	Frequency (%)
Concomitant radio-chemotherapy	66 (43.7)
Concomitant radio chemotherapy followed by surgery	22 (14.9)
Exclusive radiotherapy	12 (7.9)
Surgery followed by exclusive radiotherapy	25 (16.6)
Surgery followed by concomitant radio chemotherapy	21 (13.9)
Surgery alone	4 (2.6)
Palliative chemotherapy	1 (0.7)

Table 3, summarizes the various therapeutic modalities of different stages. Essentially our series contains 87 (63.1 %) patients with stage IIB and IIIB. These two stages are essentially treated with concomitant radio-chemotherapy.

**Table 3:** Therapeutic modalities based on clinical stage

Modalities	FIGO stage									
	IIIA	IA	IB1	IB2	IIA	IIB proximal	IIB distal	IIIB	IVA	IVB
Exclusive radiotherapy	0	0	2	0	0	0	3	5	2	0
	0,0 %	0,0 %	16,7 %	0,0 %	0,0 %	0,0 %	25,0 %	41,7 %	16,7 %	0,0 %
Concomitant radio-chemotherapy	2	0	1	7	1	18	18	16	1	1
	3,1 %	0,0 %	1,5 %	10,8 %	1,5 %	27,7 %	27,7 %	24,6 %	1,5 %	1,5 %
Surgery followed by exclusive radiotherapy	0	1	10	3	0	3	0	0	0	0
	0,0 %	5,9 %	58,8 %	17,6 %	0,0 %	17,6 %	0,0 %	0,0 %	0,0 %	0,0 %
Surgery followed by concomitant radio chemotherapy	0	0	3	4	1	0	0	0	0	0
	0,0 %	0,0 %	37,5 %	50,0 %	12,5 %	0,0 %	0,0 %	0,0 %	0,0 %	0,0 %
Concomitant radio chemotherapy followed by surgery	1	0	0	2	2	9	5	3	0	0
	4,5 %	0,0 %	0,0 %	9,1 %	9,1 %	40,9 %	22,7 %	13,6 %	0,0 %	0,0 %
Surgery alone	0	1	0	1	0	0	0	0	0	0
	0,0 %	50,0 %	0,0 %	50,0 %	0,0 %	0,0 %	0,0 %	0,0 %	0,0 %	0,0 %
Palliative chemotherapy	0	0	0	0	0	0	0	0	0	1
	0,0 %	0,0 %	0,0 %	0,0 %	0,0 %	0,0 %	0,0 %	0,0 %	0,0 %	100,0 %

**Fig. 2:**
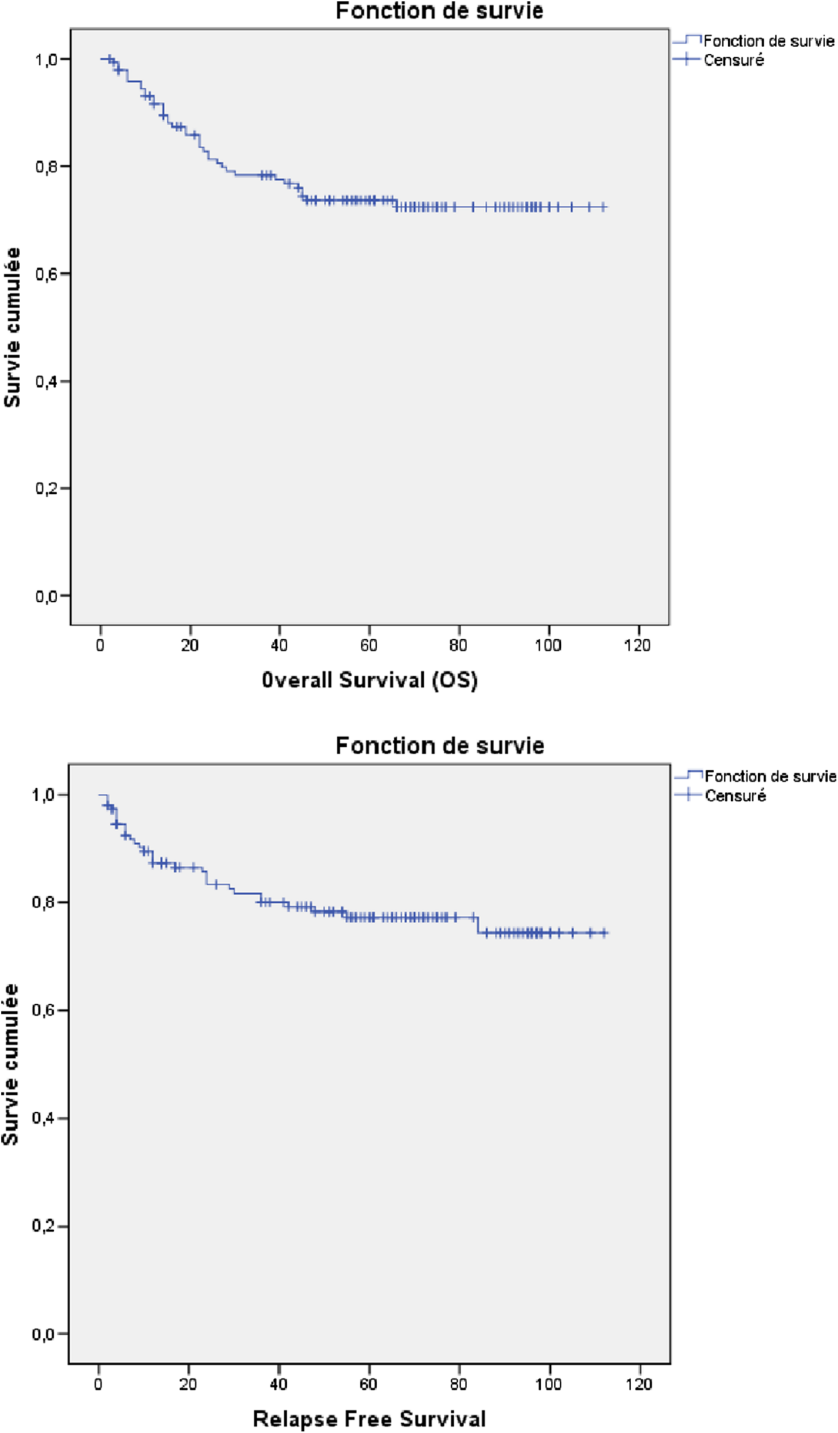
Global survival (GS) and relapse free survival (RFS)

With a mean follow-up of 51.6 months (2 to 109), we recorded 19 (12.6 %) pelvic relapse and 15 (9.9 %) metastases. The median time to onset was 19.4 months [2–33] and [34-84]. The local control rate was 63.6 % (*n* = 96) and 21 (13.9 %) patients were lost to follow-up. The overall survival (OS) at 3 years and 5 years was respectively 78.3 % and 73.6 % and the relapse-free survival (RFS) was respectively 80 % and 77.2 % (Fig. 2). We tried to contact all patients who were lost to follow-up by phone and by sending correspondence letter without resulting from response.

As shown in Table 4, univariate analysis for clinical parameters as risk factors for relapse, the only independently significant variables were the Menopausal status (*p* = 0.043) and parity (*p* = 0.010). Women with advanced cervical cancer (distal IIB, III and IV) have a higher rate of relapse 16 (25.8 %) than those with early stage disease (I, IIA and proximal IIB) but there’s statistically no significant.

**Table 4:** Univariate analysis for clinical parameters associated with the occurrence of relapse and/or metastasis

Characteristic	Pelvic relapse and/or metastases		*P* value
	Yes (*n* = 31)	No (*n* = 120)	
Age	47.97 ± 10.317	52.11 ± 11.54	0.071
Menopausal status			0.043
Premenopausal	20 (27.4 %)	53 (72.6 %)	
Postmenopausal	11 (14.1 %)	67 (85.9 %)	
Age of marriage (years)	18.57 ± 2.84	17.79 ± 3.86	0.393
Age of first pregnancy (years)	21.86 ± 4.26	19.97 ± 4.97	0.124
Parity	3.79 ± 2.04	5.19 ± 2.65	0.010
Tumor size (cm)	5.19 ± 2.01	4.62 ± 1.50	0.168
Parametrial invasion	21 (22.1 %)	74 (77.9 %)	0.596
Histological type			0.281
Squamous cell carcinoma	24 (18.8 %)	104 (81.2 %)	
Adenocarcinoma	6 (35.3 %)	11 (64.7 %)	
Pelvic adenopathy	4 (23.5 %)	13 (76.5 %)	0.658
Hemoglobin (g/dl)	11.62 ± 1.61	11.97 ± 1.88	0.448
FIGO stage			0.052
I, IIA and proximal IIB	8 (12.3 %)	57 (87.7 %)	
Distal IIB, III and IV	16 (25.8 %)	46 (74.2 %)	
Therapeutic Modalities			0.151
Concomitant radio-chemotherapy	18 (27.3 %)	48 (72.7 %)	
Radio-surgery	10 (14.7 %)	58 (85.3 %)	

Multivariate analysis was not performed for our patient because of the small number of events.

## Discussion

Cervical cancer has continued to have a devastating impact on women’s health globally, and particularly in developing countries like Morocco where it has remained the second most common cancer of women after breast cancer [10].

The demographic characteristics of the patients in this study share similarities with results from several other centers in Morocco and North Africa. For instance, the mean age of patients in this study was 51.5 (±11.5) years and the high mean parity 4.9 (±2.6) agrees with the results from other studies [14, 15]. Elsewhere in Africa, the mean age was lower: 35 years in Dakar and 48 years in Burkina Faso [16] and Madagascar [17]. The older mean age in our study probably indicates a later exposure to risk factors or reflects the belated consultation and the lack of screening. These findings affirm that menopausal status and grand multiparity were significant causal risk factor for relapse.

The presenting complaints were essentially similar to the reports in the literatures reviewed. The higher incidence of abnormal vaginal bleeding reported in literature, affirm that is an important sign in cervical cancer. Abnormal vaginal bleeding normally occurs as post-coital, inter-menstrual, or postmenopausal bleeding. Should every case of abnormal vaginal bleeding be promptly investigated, cervical cancers would be diagnosed in early stages, at a time when there could be hopes of cure. However, due to the illiteracy, poverty, ignorance, non-utilization of screening services, the majority of the women present in late stage disease [13]. Initial evaluation of lymph nodes is commonly performed with CT or RMI to minimize expense, biopsy of suspected lymph nodes was not performed because it is not a standard in cervical cancer but PET and PET/CT are the imaging modalities used to provide information for treatment decisions [18–21]. In our study, no patients have PET because we do not have a pet scan this period. Actually, since 2012, we perform a PET/CT prior to treatment to evaluate the extent of disease with particular attention to lymph node metastases to provide information to design radiation fields.

This study confirmed a delayed diagnosis of cervical cancer in Morocco. In fact, the majority of patients were presented in advanced stage (IB2-IVA) 117 (84.8 %). The stage of diagnosis is the most important independent prognostic factor [11, 12] and the survival rate at 5 years decreases with stage of diagnosis: 85 % for stage IB to 0-20 % for stage IV [22]. The mortality rate from cervical cancer is greatly dependent on stage of diagnosis. In the same way, the risk of pelvic recurrence increases with the stage, 10 % for stage IB to more than 75 % for stage IV [22, 23]. Finally, the risk of distant metastases also increases with the stage, respectively 16 %, 26 %, 39 % and 75 % for stages I, II, III and IV [24]. Early Clinical diagnosis has been responsible for the reduction of cervical cancer mortality achieved in developed countries before cervical screening programs were adopted [25–27].

Our pathological data joined those already described with the large predominance of squamous cell carcinoma [28, 29]; the most represented histological type in our study was squamous cell carcinoma 137 (86.2 %); adenocarcinomas in 18 (11.3 %) and adenosquamous cell carcinomas represented only 4 (2.5 %) of cervical cancer. Over the past 40 years, multiple reports have documented the increase in relative distribution of adenocarcinoma compared to SCC in developed countries [30, 31]. In the USA, from 1973 to 1977, the proportions of SCC and adenocarcinoma were 88 % and 12 %, respectively; however, from 1993 to 1996, the proportions were 76 % and 24 % respectively [30].

In our study, treatment varied depending on the stage of diagnosis. Radiotherapy was indicated for the different stages. For advanced tumors (≥ stage II) concomitant chemotherapy was associated with radiotherapy and /or brachytherapy which join, in general, the recommendations for the management of invasive cervical cancer [32]. Whereas surgery was a part of treatment modalities in 71 (47.7 %) patients, which 50 (33.6 %) were operated before admission to our department without multidisciplinary consultation meetings. The reasons of surgery were intricate in our patients. It was indicated for very early stages (IA and IB2) in 15 (21.1 %) cases and for patients with adenocarcinoma in 14 (19.7 %) cases. It was also performed in 21 (29.5 %) case of poor response to concomitant chemoradiotherapy. Some locally advanced cervical cancer did not receive concomitant chemotherapy because of 6 (25 %) kidney failure and 11 (45.8 %) medical comorbidities or poor performance status associated.

In our population, the rate of relapse increased with the stage of diagnosis. This association shows the importance of early diagnosis. Morocco is endowed, since March 2010, of a National Plan for the Prevention and Control of Cancer (PNPCC). Screening and treatment of cervical cancer in Morocco represent the most crucial priorities for PNPCC. A screening program for cervical cancer is being introduced as well as other measures in order to improve the opportunities of access to early diagnosis by reducing geographical obstacles, multiply the number of centers of diagnosis confirmation, reduce economic barriers and provision facilities and resources. The installation of a screening program for cervical cancer will be based on infrastructure and personnel, primarily general practitioners, of primary level of health care delivery. A pilot mass screening performed in Lyon has clearly shown that intensive action, involving all local stakeholders including general practitioners, could reach a population of women who do not benefit from an adequate gynecological following-up [33].

We conducted our study in radiotherapy department of Military Hospital Mohamed V between January 2005 and February 2010. Despite recruits a small proportion of cancer cases, our center is accounted the one of public centers of cancer management in Morocco and we believe that our population can be considered as a representative sample of cases of cervical cancer who access to health system in Morocco.

## Conclusion

Cervical cancer most often afflicts women in developing countries. Our study confirmed a delayed diagnosis of cervical cancer in Morocco and affirmed that menopausal status and grand multiparity were significant causal risk factor for relapse. As in the literature, advanced stages are essentially treated with concomitant radio-chemotherapy.

Despite the limitation of our study, the results may represent an important tool in guiding the actions and measures of early diagnosis of cervical cancer in Morocco.
